# Supplementary material for: Influencing factors and configuration pathways of fundraising effectiveness in charitable foundations: An FSQCA analysis of 54 cases in China
Source: PLoS One. 2026 Jun 3;21(6):e0349317. doi: 10.1371/journal.pone.0349317 (PMC13232945; doi:10.1371/journal.pone.0349317)
Supplement: Attachment — Submitted filename: Response to Reviewers.docx [file pone.0349317.s002.docx]

Response to Reviewers

Manuscript title: Influencing Factors and Configuration Pathways of Fundraising Effectiveness in Charitable Foundations: An FSQCA Analysis of 54 Cases in China

Manuscript ID: [PONE-D-25-66814]

Dear Editor and Reviewers,

Thank you very much for your letter and for the reviewers’ insightful comments concerning our manuscript entitled “Factors and Configuration Pathways of Fundraising Effectiveness in Charitable Foundations: An FSQCA Analysis of 54 Cases in China” (Manuscript ID: [PONE-D-25-66814]). These suggestions are extremely valuable and helpful for improving the quality of our paper. We have carefully considered all the comments and revised the manuscript accordingly.

In addition, several figures (figure1, figure6) in the manuscript have been updated and optimized for better presentation and clarity. And we have also added four new figures (figure2, figure3, figure 4, figure 5). The original figure files have been removed from the submission system, and only the revised figures are included in the revised manuscript.

Enclosed please find the revised manuscript, along with a version showing all changes via track changes, and this point-by-point response to the reviewers.

We hope that the revisions meet with your approval and that the manuscript is now suitable for publication in PLOS ONE.

Thank you again for your time and effort.

Sincerely,

Heran Zou

Wuhan Institute of Technology

**Response to Reviewer 1**

**Comment 1:** Construct validity problems: several key variables do not measure what the manuscript claims.

**Response:** We greatly appreciate the reviewer for pointing out the critical issue of construct validity regarding our key variables. We fully agree that the original operational definitions and measurements of Fundraising Effectiveness (FE), Brand Building Level (BBL), and Media Supervision (MS) were inconsistent with their theoretical connotations, which may lead to misunderstanding and biased interpretation. We have carefully revised the conceptual definition, operational measurement, variable naming, and theoretical logic in the revised manuscript to ensure consistency between construct and measurement. Detailed revisions are addressed point-by-point below.

**Comment 1.1:** Fundraising Effectiveness (FE) is defined as “aggregate revenue generated through project activities and providing public goods/services” (Methods 3.2.2; Table 1). This is not clearly “fundraising effectiveness” as commonly understood. It mixes: fundraising income vs program/service revenues, scale vs efficiency, and possibly government/purchased services if booked as project income.
If the construct is “fundraising effectiveness,” the outcome should be operationalized as: total donations received; public fundraising revenue; online donation amount; or a ratio-based metric (e.g., fundraising revenue per staff, per project, or as share of total revenue). At minimum, you must clarify the accounting category used in annual reports (which line item) and justify why it maps to “fundraising effectiveness.”
**Response:** We sincerely thank the reviewer for this insightful comment. We agree that the original definition mixed fundraising income, program revenue, scale, and efficiency, which does not match the common understanding of fundraising effectiveness.

In the revised manuscript, we have re-defined Fundraising Effectiveness strictly as donation-related revenue rather than total project income; clearly specified the accounting line item from the annual reports used for measurement and justified why this indicator represents fundraising effectiveness in the context of Chinese non-profit organizations. The revised sentence now reads as follows: The outcome variable is Fundraising Effectiveness (FE), defined as the total annual public donation revenue of charitable foundations (excluding program service income, government purchase service income and other non-fundraising income). The data is extracted from the "donation income" line item in the annual financial reports of foundations, which strictly reflects the actual fundraising capacity of organizations (consistent with the universal definition of fundraising effectiveness in nonprofit research). And in Table 1, we have changed the measurement dimension of FE to “Total annual public donation revenue of charitable foundations”.

**Revisions in the Manuscript:** These revisions have been made in Section 3.2.2 and Table 1 to ensure conceptual rigor.

**Comment 1.2:** “Brand Building Level (BBL)” is measured as the “number of public welfare and charitable projects undertaken this year.”
This is closer to program activity volume/scale than brand building. Using project count as “brand building” risks circularity: more projects can mechanically generate more “project revenue” if FE is tied to program activities. That can create an artificial configuration where “BBL” predicts “FE” because both reflect activity scale.
Suggested fix: either re-label BBL as “program activity intensity/portfolio breadth” and adjust theory, or construct a genuine brand proxy: brand mentions, media exposure metrics, third-party ratings, social media followers/engagement, donor retention, website traffic, or reputational awards.
**Response:** We sincerely appreciate the reviewer’s critical comment. We fully agree that using the number of projects mainly reflects program scale rather than brand building and may lead to spurious correlation with Fundraising Effectiveness. Following the reviewer’s suggestion, we have removed the original Brand Building Level (BBL) construct and replaced it with Program Activity Intensity (PAI) in the revised manuscript. At the same time, we adjusted the theoretical framework and research hypotheses accordingly, as well as other areas where BBL has appeared in this study, to ensure consistency between concepts and logic.

According to the reviewer's comments, the original text is now revised as follows: revise section 2.2 to: In this context, program activity intensity and professional management emerge as key organizational capabilities. Sufficient program activities act as a signal to effectively reduce information asymmetry faced by donors, strengthen organizational brand building, and enhance their trust and willingness to donate [24]. Waters (2011) found that effective relationship management with non-profit organizations significantly increases donor loyalty and willingness to give [25]. Additionally, establishing political connections with key resource holders, such as the government, has been proved to be an effective way in acquiring critical resources and legitimacy, particularly in environments with high institutional uncertainty [26].”

Revise section 2.4 to: “Organizational Dimension. This dimension incorporates discussions on professional management and program activity from the “organizational capacity” literature. This dimension focuses on the characteristics and abilities that can be controlled or constructed within the organization. In the highly competitive charitable market, foundations must rely on intrinsic capabilities to earn trust. Drawing on resource dependence theory, foundations must develop internal core competencies to manage resource channels. Professional Management Level (measured by full-time staff count) reflects an organization’s human resource foundation and operational effectiveness, which determines its ability to translate resources into social impact [36]. Program Activity Intensity (measured by the number of projects) is a strategic asset for organizations to carry out business activities, send operational signals to the public, and establish stable connections with donors [24], which helps to manage external resource dependence. Therefore, this study selects Professional Management Level (PML) and Program Activity Intensity (PAI) as the core variables to measure the organizational dimension.

**Revisions in the Manuscript:** These revisions have been implemented in Sections 2.2 and 2.4, as well as in Figures 1 and 6, and across all other instances of BBL throughout the manuscript.

**Comment 1.3:** “Media Supervision (MS)” is operationalized as a 2–4 score based on existence of website/WeChat/Weibo and information disclosure.

This is not media supervision; it is digital presence + disclosure practice, and it is largely an organizational capability rather than external supervision. The paper’s interpretation (MS as an external constraint) conflicts with its measurement (internal adoption).

Suggested fix: rename to “digital transparency infrastructure” (or “information disclosure capacity”), and if you truly want “media supervision,” measure negative/positive media coverage, scrutiny events, or exposure to investigative reporting.

**Response:** We thank the reviewer for identifying this important inconsistency. The original measurement actually reflected digital transparency and information disclosure capacity, rather than external media supervision.

We have revised the variable name to Digital Transparency Infrastructure and adjusted its theoretical interpretation accordingly. This adjustment ensures consistency between theoretical definition and empirical measurement.

The revision can be found in Section 2.4. Section 2.4 has been modified as follows：Technological Dimension. This dimension creatively transforms the micro mechanism of transparency, trust and technology adoption in the “donor trust” literature into an organization’s strategic capacity to proactively employ information and communication technologies for information disclosure and trust-building. The term “technology” herein is construed in a broad sense, encompassing not only instrumental technologies such as digital payment, data analysis, and blockchain traceability, but also information communication approaches, digital operation platforms, and standardized disclosure procedures adopted by organizations to adapt to the digital philanthropy context and enhance fundraising efficiency. In the digital era, information disclosure and communication technologies constitute the core infrastructure for establishing the credibility of charitable foundations, which directly affects the formation and maintenance of donor trust. Existing research has verified that donor trust under the framework of “Internet + Public Welfare” is highly dependent on the transparency of charitable information and the traceability of fund utilization. Given data availability and compatibility with the research sample, this study adopts Digital Transparency Infrastructure (DTI) as the core measurement indicator of the technological dimension, defining it as the behavior of foundations in conducting proactive and standardized information disclosure through multiple channels including official websites, WeChat official accounts, and Sina Weibo. In essence, DTI captures the capability of foundations to adopt and apply modern information and communication technologies, proactively establish information transparency, respond to public concerns, and transmit credible signals to donors. Information disclosure behaviors under this dimension are no longer merely external supervision constraints, but rather “trust-building technologies” that foundations must master and actively employ in the digital philanthropy ecosystem, representing the core subset of the broad technological dimension that is most directly associated with fundraising efficiency and quantifiable with empirical data. This study takes the information communication and disclosure technologies underpinning Digital Transparency Infrastructure as the core manifestation of the technological dimension, leaving room for future research to expand the measurement scope of the technological dimension.

**Revisions in the Manuscript:** These revisions have been implemented in Section 2.4, Figures 1 and 6, and all other instances where MS is referenced throughout the manuscript.

**Comment 2:** Calibration is internally inconsistent; GRA thresholds appear invalid and will distort results
Table 3 sets GRA calibration as Fully in = 150, Intersection = 0, Fully out = 0. This is not a proper three-anchor calibration because the cross-over and full-out are identical (0). With many zeros in GRA (Table 2 min=0), this will force a large mass of cases to extreme membership and can create spurious necessity/sufficiency patterns.
Moreover, the text states anchors are set at 25/50/75 percentiles (Garcia-Castro & Francoeur 2016), but the GRA anchors do not look like 25/50/75 percentiles given mean 2785 and max 100000. This suggests either (i) misreporting, or (ii) using a different rule only for GRA.
Required revision:
1) Report the empirical 25th/50th/75th percentiles for each raw variable and show how anchors were chosen.
2) Consider log-transforming highly skewed monetary variables before calibration.
3) Use a nonzero cross-over for GRA (e.g., median) and distinct full-out (e.g., 10th/25th percentile), or adopt theoretically justified anchors.
**Response:** We are very grateful to the reviewer for this rigorous and insightful comment on the calibration procedure in QCA. We fully agree that the original calibration thresholds for GRA were improperly specified, with identical values for the crossover and full-out anchors, which is not consistent with standard QCA practice and may produce misleading results. In addition, we acknowledge the inconsistency between the reported percentile‑based calibration rule and the actual thresholds used for GRA.

Following the reviewer’s requirements, we have revised the calibration as follows: 1. We now report the 25th, 50th, and 75th percentiles for all raw variables, and clearly document how each calibration anchor was determined. 2. We reset the GRA calibration thresholds with a nonzero crossover point (median) and a distinct full-out anchor (25th percentile), all based on empirical distribution and consistent with the percentile method applied to other variables. Regarding the suggestion of log-transformation, we appreciate this advice. Since QCA calibration already transforms raw data into 0–1 fuzzy scores and accommodates non-linear relationships, we believe the calibration procedure sufficiently mitigates the influence of skewed variables. We thus retain the original raw variable while ensuring all calibration thresholds are consistently based on percentiles and clearly reported.

We have revised section 3.3 to: Considering practical situations, the study primarily employs fuzzy calibration. At the same time, to ensure relatively objective calibration, the direct calibration method was adopted to perform quartile calibration on the antecedent conditions, following the approach of Garcia Castro and Francoeur (2016). The study used fsQCA3.0 software to select the upper quartile (75%), median (50%), and lower quartile (25%) as the three anchor points for Fully in, Intersection Point, and Fully out [51]. It is worth noting that during the calibration of the conditional variable GRA, it was found that due to the influence of the distribution characteristics of the sample data, the intersection values set at the 50% percentile were set to 0, resulting in numerical overlap between the intersection points and Fully out points, making it difficult to form effective set discrimination. If the quantile method continues to be used, it may weaken the identification of the result variables in fuzzy set partitioning, which is not conducive to the stability of subsequent configuration analysis. Therefore, while maintaining the overall idea of quantile calibration, this article uses the mean as an auxiliary intersection point for calibration to ensure that the result set has a clear boundary between Fully in and Fully out. And we adjusted the calibration points of GRA in Table 3. These revisions improve the reliability of calibration and the robustness of QCA results. In addition, I also modified the calibration points of DTI variables with calibration errors in Table 3.

**Revisions in the Manuscript:** These revisions have been made in Section 3.3 and Table 3.

**Comment 3:** Limited diversity and quasi-constant conditions: MS has minimum 2 and very low variance
Descriptives show MS ranges from 2 to 4, mean 2.87, SD 0.73, min 2. This implies most cases already satisfy “some MS,” and no case is truly “low MS” in raw terms. Yet the necessity test claims “Strong media supervision” is necessary for high FE (consistency 0.908).
If MS is near-constant, it can appear necessary simply because it rarely varies and is present for almost everyone. This is a classic QCA pitfall: necessity can be an artifact of low variance.
Required revision:
1) Provide the distribution of raw MS scores and calibrated memberships (histogram or frequency table).
2) Test whether the necessity claim holds under alternative MS calibration anchors.
3) Consider replacing MS with a more discriminating measure (e.g., actual disclosure quality indices; frequency of updates; third-party transparency ratings).
**Response:** We sincerely appreciate the reviewer for identifying this key methodological issue in QCA. We fully agree that the original Media Supervision (MS) variable has a narrow range (2–4) and low variance, which represents a typical quasi‑constant condition. As the reviewer correctly pointed out, the significant necessity result for MS may be an artifact of limited diversity rather than a substantive theoretical relationship.

To address this problem, we have implemented the following revisions: 1. We provide the full distribution of the raw MS scores and calibrated fuzzy membership scores frequency tablein the revised manuscript in Figure 2 and 3. 2. According to the fifth comment from the reviewer, we have added a scatter plot of the Digital Transparency Infrastructure (formerly MS). Based on the scatter plot, it has been confirmed that DTI (formerly MS) is no longer a necessary condition for FE, so there is no need to test whether the necessity statement is valid under the alternative MS calibration anchor. 3. According to the reviewer's comment 1.3, we have replaced MS with Digital Transparency Infrastructure (DTI). Section 3.3 has been modified as follows: Notably, the raw scores of the Digital Transparency Infrastructure (DTI) variable range from 2 to 4, with a mean of 2.87 and a standard deviation of 0.73. All sample foundations exhibit a certain level of digital transparency infrastructure, with no cases of complete absence. Figure 2 presents the distribution of the raw DTI scores, and Figure 3 displays the distribution of the calibrated fuzzy-set membership scores of DTI. As shown in Figure 3, the calibrated DTI membership scores exhibit a bimodal polarization: 18 cases have a membership score ≤ 0.2, and 36 cases have a membership score ≥ 0.8, with no observations in the intermediate range. This distribution pattern implies that DTI approximates a quasi-necessary condition in the sample, which may lead to a high consistency score in univariate necessity analysis. However, this is more likely to reflect a limitation of the sample structure rather than indicating that DTI independently constitutes a necessary condition for high fundraising income. Accordingly, this study focuses more on the joint effects of DTI with other factors within configurational conditions, rather than its independent explanatory role in terms of necessity. Histograms of the raw DTI scores and calibrated fuzzy‑set membership scores of DTI are presented in Figures 2 and 3, respectively.

**Revisions in the Manuscript:** These revisions have been made in Section 3.3 and Figure 2 and Figure 3

**Comment 4:** Truth table construction and labeling issues suggest errors in analysis pipeline
In Table 5 (Truth Table), the columns list PC GML PMl BPL MS but earlier you define GRA and PML; “GML/PMl” appear inconsistent typos.
Additionally, Table 5 shows FE=0 for several rows even when MS=1 and PC=1 etc., but the manuscript then states it uses the “complex solution” to exclude counterfactuals. The procedure needs to be described precisely: frequency threshold (how many cases per row retained), consistency threshold for sufficiency, PRI consistency (to avoid simultaneous subset relations).
Required revision:
1) Provide the full fsQCA settings: frequency cutoff, consistency cutoff, PRI cutoff, directional expectations (intermediate solution), and how contradictions were handled.
2) Include the intermediate and parsimonious solutions (not only complex), because “core vs peripheral” is defined relative to parsimonious/intermediate comparisons. The current presentation risks misclassifying “core” conditions.
**Response:** We greatly appreciate the reviewer for carefully identifying the labeling errors and incomplete description of the fsQCA analytical procedure. We apologize for the typographical mistakes in variable abbreviations (GML/PMl) in Table 5, which have now been corrected to GRA and PML to ensure full consistency with the definitions provided earlier in the manuscript. Following the reviewer’s requirements, we have comprehensively supplemented and standardized the fsQCA analysis settings in the revised manuscript:

1. We clearly report the frequency cutoff, consistency threshold, and PRI consistency threshold used for truth table construction, as well as the handling of contradictory rows and directional expectations for the intermediate solution. Section 4.2 has been revised as follows: Prior to conducting the configurational analysis, a truth table was constructed using fsQCA 3.0 software (Table 5). Given the relatively large number of condition variables in this study and the varying frequencies of different condition combinations across the sample, the indiscriminate inclusion of all combinations may introduce a large number of logical remainders lacking empirical support, thereby undermining the robustness and explanatory power of the configurational results. Therefore, it is necessary to reasonably set the thresholds for case frequency and consistency. Considering the total sample size of 54 in this study and following conventional practices in existing fsQCA studies for medium-sized samples, the case frequency threshold was set to 1 to maximize the retention of sample information and avoid the loss of valid configurations. For the consistency threshold, the academic community generally agrees that consistency should not be lower than 0.75, and higher values indicate stronger sufficiency of the condition configurations for the outcome variable. Integrating the theoretical expectations and sample characteristics of this study, and referring to relevant literature, the raw consistency threshold was set to 0.8, and the PRI consistency threshold was set to 0.7 [52]. This setting helps to eliminate simultaneous subset relations between configurations, avoid interpretive biases caused by asymmetric causal relationships, and improve the rigor of the analytical results.

2. We now present three solutions: complex, intermediate, and parsimonious, and clearly distinguish core conditions and peripheral conditions based on the comparison between the parsimonious solution and intermediate solution, in accordance with standard fsQCA procedures. Revise section 4.2 in this regard as follows: According to the truth table for QCA data statistics, Fiss (2011) uses solid circles to represent the presence of conditions and hollow circles to represent the absence of conditions in order to visually report the results [46]. Circle size distinguishes core conditions from peripheral conditions, while blank spaces indicate optional conditions. Core conditions are defined as those that appear in both the parsimonious solution and the intermediate solution and are critical for the outcome. Peripheral conditions refer to those that appear only in the intermediate solution in the QCA analysis and are less important for the outcome compared with core conditions.The complex solution presents the most conservative configurational relationships. The intermediate solution, which incorporates limited counterfactuals based on theoretical expectations, serves as the primary interpretive basis in this study.

**Revisions in the Manuscript:** These revisions have been made in Section 4.2.

**Comment 5:** Interpretation problems: “necessary” vs “sufficient,” and “core” claims are overstated
You assert that MS is a necessary condition for high FE because consistency >0.9 (0.908). However, standard practice is to also examine: whether necessity holds for the negated outcome (it appears MS is also high for low FE: 0.923), which undermines practical necessity, relevance-of-necessity metrics (RoN) and empirical coverage logic, and trivial necessity due to high prevalence.
In Table 4, “Strong media supervision” has consistency 0.908 for high FE but also 0.923 for low FE. This is a red flag: a condition that is “necessary” for both outcome and non-outcome is not discriminating; it may simply be ubiquitous.
Also, the manuscript claims PML, BBL, and GRA are “sufficient but not necessary” based on their necessity-table consistency exceeding 0.8. That is not a valid inference: a necessity-table consistency >0.8 does not establish sufficiency; sufficiency must be evaluated using sufficiency analysis/truth table solutions for X→Y, not by necessity metrics.
Required revision:
1) Separate necessity analysis from sufficiency analysis properly.
2) Add XY-plots for key necessity claims.
3) Avoid “core element” language unless the core/peripheral status is methodologically supported and robust to specifications.

**Response:** We sincerely thank the reviewer for this highly professional comment on the distinction between necessity and sufficiency in QCA. We fully agree that the previous interpretation was inappropriate and may have led to misleading conclusions. We have carefully revised all relevant descriptions in accordance with standard QCA rules.

Specific revisions are as follows:1. We have strictly separated necessity analysis and sufficiency analysis, and no longer infer sufficiency based on necessity consistency scores. We only interpret necessity results based on necessity analysis, and sufficiency only based on truth table and sufficient condition analysis. 2. We have added XY-plots for key necessary condition tests to visually demonstrate the necessity relationship and strengthen the rigor of the analysis. 3. We have removed overstatements regarding “necessary conditions” and “core elements” and only use these terms when they are fully supported by empirical results and robust across different calibration specifications.

In addition, we acknowledge that a condition with high consistency for both high FE and low FE lacks discriminative power. We have revised the interpretation of media supervision (MS) accordingly and no longer claim it as a meaningful necessary condition due to its high prevalence and low discrimination.

We have revised 4.1 as follows: Consistency measures the sufficient and necessary relationship between conditional variable X and outcome variable Y in geometric membership, while coverage measures the extent to which these given conditions or combinations of conditions explain the occurrence of the outcome. Ragin (2008) posits that consistency values range from 0 to 1, with a consistency score above 0.9 and coverage is greater than 0.5 indicating that the conditional variable is a necessary condition for the outcome [45]. Concurrently, if a single variable constitutes a sufficient condition for the outcome variable, its fuzzy set score must be less than or equal to that of the outcome variable, with a consistency index exceeding 0.8.

As shown in the results of single-factor necessity tests in Table 4, only strong Digital Transparency Infrastructure (DTI) presents a consistency score above 0.9 and coverage above 0.5, suggesting that strong DTI may be a necessary condition for foundations to achieve high fundraising income. In contrast, high professional management capability (0.882), high program activity intensity (0.877), and strong government resource acquisition capacity (0.832) all exceed the threshold of 0.8, indicating that these four variables represent sufficient but not necessary conditions for variations in fundraising performance among charitable foundations. However, the consistency values of all other variables are below 0.75, implying that these variables cannot independently explain changes in fundraising income. This necessitates configurational analysis to identify combinations of multiple factors driving variations in fundraising income.

In fsQCA, consistency ≥ 0.9 and coverage > 0.5 are commonly used as criteria for identifying a necessary condition, while consistency ≥ 0.8 serves as a reference threshold for assessing the sufficiency of a single condition. It should be emphasized that valid judgments of sufficiency rely on truth table and configurational analysis rather than single-condition consistency alone, which constitutes the core distinction between necessity and sufficiency analyses. As shown in Table 4, strong DTI yields a consistency score of 0.908 for high fundraising performance, which meets the numerical benchmark for a necessary condition. However, its consistency for low fundraising performance reaches 0.923, with no significant difference between the two values. This indicates that DTI lacks discriminatory power between high and low fundraising performance, requiring further verification of its necessity via visual analysis.

To further examine the necessity of individual conditions, this study presents XY plots of necessity analyses for DTI with respect to high fundraising performance (Y) and low fundraising performance (~Y) (Figures 2 and 3). A core criterion for identifying a necessary condition in fsQCA is that the fuzzy-set membership of the condition is always less than or equal to that of the outcome, which appears as a concentration of observations in the upper-left region above the 45° diagonal in XY plots.

As shown in Figure 2, observations for DTI relative to high fundraising performance do not cluster noticeably in the upper-left region; instead, many points lie near the vertical Y-axis, thus failing to satisfy the distributional requirement for a necessary condition. Similarly, Figure 3 reveals no upper-left clustering for DTI relative to low fundraising performance. Combined with the results in Table 4, DTI yields a consistency of 0.908 for high fundraising performance and 0.923 for low fundraising performance, with the latter even slightly higher. This demonstrates that DTI is prevalent across the sample and lacks discriminatory power between high and low fundraising outcomes and therefore is not a necessary condition for achieving high fundraising performance.

Furthermore, no other single condition reaches the 0.9 consistency threshold for necessity, and single-condition consistency cannot be used to infer sufficiency. Accordingly, this study does not identify any single condition as either necessary or sufficient for high fundraising performance. Instead, all conditions are treated as potential explanatory variables and incorporated into subsequent configurational analysis to explore their combined effects.

The XY plots for the necessity analysis of Digital Transparency Infrastructure (DTI) with respect to high fundraising performance (Y) and low fundraising performance (~Y) are presented in Figures 4 and 5, respectively.

**Revisions in the Manuscript:** These revisions have been made in Section 4.1 and Figure 4 and Figure 5. Section 4.3 and the abstract section have been revised with relevant expressions.

**Response to Reviewer 2**

**Comment 1:** - Narrow Variable Definition: The "Technological Dimension" in the TOE framework is operationalized almost exclusively as "media supervision". Critics may argue that "technology" should encompass broader infrastructure, such as digital payment systems or data analytics, rather than just information disclosure behavior
**Response:** We sincerely appreciate the reviewer’s constructive comment on the Technological dimension in the TOE framework. We agree that the original presentation was too narrow and may give the impression that the Technological dimension was operationalized only as media supervision. In the revised manuscript, we have substantially expanded the conceptual connotation of the Technological dimension within the TOE framework. We clarify that this dimension reflects broader digital technology infrastructure and capability, including digital operation, information disclosure, online communication, and digital transparency practices. Notably, we retain the original core measure of Digital Transparency Infrastructure (DTI) throughout the empirical analysis, as DTI appropriately captures the key digital technology attributes relevant to this study. We have strengthened the theoretical justification for using DTI as the proxy for the Technological dimension, to better align measurement with the expanded conceptual definition.

These revisions are presented in Section 2.4 of the revised manuscript. The specific modifications are as follows: Technological Dimension. This dimension creatively transforms the micro mechanism of transparency, trust and technology adoption in the “donor trust” literature into an organization’s strategic capacity to proactively employ information and communication technologies for information disclosure and trust-building. The term “technology” herein is construed in a broad sense, encompassing not only instrumental technologies such as digital payment, data analysis, and blockchain traceability, but also information communication approaches, digital operation platforms, and standardized disclosure procedures adopted by organizations to adapt to the digital philanthropy context and enhance fundraising efficiency. In the digital era, information disclosure and communication technologies constitute the core infrastructure for establishing the credibility of charitable foundations, which directly affects the formation and maintenance of donor trust. Existing research has verified that donor trust under the framework of “Internet + Public Welfare” is highly dependent on the transparency of charitable information and the traceability of fund utilization. Given data availability and compatibility with the research sample, this study adopts Digital Transparency Infrastructure (DTI) as the core measurement indicator of the technological dimension, defining it as the behavior of foundations in conducting proactive and standardized information disclosure through multiple channels including official websites, WeChat official accounts, and Sina Weibo. In essence, DTI captures the capability of foundations to adopt and apply modern information and communication technologies, proactively establish information transparency, respond to public concerns, and transmit credible signals to donors. Information disclosure behaviors under this dimension are no longer merely external supervision constraints, but rather “trust-building technologies” that foundations must master and actively employ in the digital philanthropy ecosystem, representing the core subset of the broad technological dimension that is most directly associated with fundraising efficiency and quantifiable with empirical data. This study takes the information communication and disclosure technologies underpinning Digital Transparency Infrastructure as the core manifestation of the technological dimension, leaving room for future research to expand the measurement scope of the technological dimension.

**Revisions in the Manuscript:** These revisions have been made in Section 2.4.

**Comment 2:** - The study uses 54 cases, which the authors justify as a "medium sample size" optimal for QCA. However, they acknowledge that this size leaves a significant portion of explanatory variables unobservable, which could be more explicitly addressed as a limitation in the conclusion.

**Response:** We thank the reviewer for this careful remark. Although 54 cases are generally recognized as appropriate for fuzzy-set QCA, we acknowledge that the sample size still restricts the number of conditions included and may limit the generalizability of the findings. In the revised manuscript, we have explicitly incorporated this issue into the limitations section and provided a clear and objective statement about the constraints imposed by the sample size, so that readers can better interpret the results.

These revisions are presented in Section 5.2 of the revised manuscript. The specific modifications are as follows: This study has several limitations. First, in terms of sample size, this study adopts a medium sample design consisting of 54 cases, which is consistent with the recommended sample range for fsQCA research. However, the relatively limited sample restricts the observation of some potential explanatory variables, including micro-level organizational operational practices, regional philanthropic culture, and the depth of digital platform application. Therefore, this study may not fully capture the heterogeneity in fundraising efficiency among different foundations, nor does it comprehensively examine the interplay between contextual factors and configurational paths. Second, regarding variable coverage, the present study operationalizes the technological dimension using information disclosure practices. Although this is consistent with data availability, it narrows the concept of “technology” to a subset of digital tools. Future research should incorporate a broader range of digital technologies, such as artificial intelligence-driven donor matching, blockchain traceability, and data analytics-enabled fundraising optimization, to evaluate the impact of technology more comprehensively. Third, this study focuses on national-level charitable foundations. Given the pronounced Matthew effect in China’s philanthropic sector (i.e., resources are concentrated toward mature foundations), the research findings may not be fully generalizable to local foundations. Future studies should expand the sample to include municipal- and county-level foundations to explore cross-level configurational differences.

**Revisions in the Manuscript:** These revisions have been made in Section 5.2.

**Comment 3:** - Contextual Depth on the "Matthew Effect": The paper mentions a significant "Matthew effect" (where successful organizations attract even more resources) in Chinese foundations but does not fully integrate how this systemic bias might influence the "causal asymmetry" found in the fsQCA results.

**Response:** We sincerely appreciate the reviewer’s professional and constructive suggestion on strengthening the contextual integration between the Matthew effect and causal asymmetry in fsQCA analysis. We fully agree that clarifying the intrinsic linkage between the systemic bias of the Matthew effect and the causal asymmetry identified in this study can significantly enhance the theoretical depth and interpretive rationality of the paper. In response to this comment, we have comprehensively revised and supplemented the relevant content in Section 1 (Introduction) and Section 4.3 (Discussion) of the manuscript. Specifically, in Section 1, we explicitly clarify the theoretical connection between the Matthew effect as a systemic resource allocation bias and the causal asymmetry of fundraising efficiency in Chinese charitable foundations, explaining how the Matthew effect leads to unbalanced resource distribution and further shapes the asymmetric causal characteristics of "convergence in high-efficiency paths and divergence in low-efficiency paths". In Section 4.3, we further elaborate on the formation mechanism of causal asymmetry from the perspective of the Matthew effect: under the influence of this systemic bias, foundations with low fundraising performance fall into a vicious cycle of resource shortage, and their core shortcomings (e.g., insufficient program activity intensity, weak professional management) cannot be compensated by single conditional improvements, resulting in a configurational logic completely different from that of high-fundraising-performance foundations. We also emphasize that the Matthew effect reinforces the non-corresponding relationship between conditions leading to high fundraising returns and those causing low fundraising returns, which is the essential institutional reason for the causal asymmetry in fsQCA results.

These revisions are presented in Section 1 and Section 4.3 of the revised manuscript. The specific modifications are as follows: （Section 1）Currently, although the role of charitable foundations is increasingly prominent and their funding scale continues to grow, the development of charitable foundations in China has shown a significant “Matthew effect” in their development process [4]. As a systemic bias in the development of charitable foundations, the Matthew effect leads to the concentration of resources in top-tier foundations, while grassroots foundations fall into fundraising difficulties. This unbalanced development is inherently linked to the “causal asymmetry between high and low fundraising efficiency” identified in this study: the core advantages of top-tier foundations (intensity of program activities, digital transparency infrastructure) further strengthen their resource acquisition capacity, whereas the shortcomings of grassroots foundations (low intensity of program activities, inadequate professional management) are amplified by the Matthew effect, ultimately forming the causal asymmetry characterized by “convergence in high-efficiency paths and divergence in low-efficiency paths.” The vast majority of organizations are deeply trapped in fundraising challenges, with many struggling with low fundraising effectiveness and unstable funding sources, which severely constrain their organizational growth and social functions [5-6]. In practice, charitable foundations often remain dependent on traditional paths, revealing deep-seated problems such as “emphasizing scale expansion over efficiency,” “prioritizing resource acquisition over brand maintenance,” and “weak public trust.” （Section 4.3）The results reveal that low fundraising returns in charitable foundations are also influenced by the combined effects of political and external oversight dimensions. Different combinations of political connections with digital transparency infrastructure, professional management levels, and program activity intensity not only do not lead to low fundraising returns but can also explain high fundraising effectiveness. Therefore, analyzing the combined effects of various influencing factors is essential when considering fundraising returns in charitable foundations. Simultaneously, the findings confirm that under the logical premise of “causal asymmetry”, the conditions leading to high fundraising returns for charitable foundations do not correspond one-to-one with those causing low fundraising returns, indicating asymmetry. The formation of this asymmetric feature is closely associated with the systematic resource allocation bias caused by the Matthew effect in the charitable sector. Under the influence of the Matthew effect, foundations with low fundraising performance are highly prone to falling into a vicious cycle: although such foundations may also possess the basic conditions of strong political connections and robust digital transparency infrastructure, their critical shortcomings—insufficient program activity intensity or weak professional management level—prevent them from translating existing conditions into actual fundraising capabilities. The persistent downturn in fundraising performance further deprives foundations of critical resources such as capital and talent to strengthen program activities and improve professional management. This ultimately results in a configurational logic distinct from that of high-fundraising-performance foundations. It also indicates that merely adjusting a single condition in reverse cannot effectively resolve the development dilemma of low fundraising performance among charitable foundations.

**Revisions in the Manuscript:** These revisions have been made in Section 1 and Section 4.3.

**Comment 4:** - IMHO it is also necessary to suggest possible development paths in the Conclusions section.

The development paths could possibily focus on expanding the "Technology" dimension, transitioning to longitudinal data analysis, and investigating the heterogeneity of foundation types beyond the national level. For example, based on "Matthew effect" and the current sample limitation the following research opportunities could be identified:

-- conceptual expansion if the Technological Dimension through adoption of AI-driven donor matching, blockchain for fund traceability, and big data analytics and exploring how interactive experiences during the donation process—rather than just information disclosure—impact loyalty and perceived efficacy,

-- transition to temporal and dynamic analysis of timeshifts of different pathways esp. highly effective pathways,

-- comparison of causal paths for success for National-wide and local foundations,

-- investigation of difference in roles played by political connections: governmental-linked foundations vs. purely private initiatives,

-- assymetry of failure investigation e.g. to help fundation managers to avoid it.

There is so much to do in the future. :)

**Response:** We would like to express our sincere gratitude to the reviewer for these insightful and forward-looking suggestions. We fully agree that enriching the Future Research Directions section is crucial to address the current limitations and extend the theoretical contributions of the study. In accordance with the reviewer’s advice, we have systematically revised and expanded the Section 5.3 (Future Research Directions) in the revised manuscript. Specifically, we have incorporated all five research pathways proposed by the reviewer, organizing them into a logical framework that aligns with the core findings of our study. The detailed modifications are summarized as follows:

5.3 Future Research Directions: First, expand the technological dimension. This study only takes digital transparency infrastructure as the core of the technological dimension, focusing on the impact of foundations’ information disclosure capacity on fundraising performance. Against the backdrop of digital philanthropy development, technological applications such as artificial intelligence-driven donor matching, blockchain-enabled fund traceability, and big data analytics-empowered precise fundraising are profoundly reshaping the operational model of charitable fundraising and donor experience. Follow-up studies can incorporate such intelligent technology applications into the analytical framework, explore their influencing paths on donor interactive experience, and the internal mechanism through which interactive experience further acts on donation loyalty and perceived donation effectiveness. In addition, future research can excavate new configurational paths for technology-enabled improvement of fundraising performance of charitable foundations to adapt to the development needs of philanthropy in the digital era.

Second, adopt longitudinal and dynamic analyses. This study adopts a cross-sectional fsQCA design, which limits the exploration of temporal dynamics. Future research should conduct longitudinal tracking to analyze the evolution of configurational paths at different developmental stages, as well as the temporal changes in the relative importance of core conditions. Dynamic analysis can enhance the explanatory power of the formation mechanism of long-term fundraising efficiency.

Third, compare national and local foundations. To address the Matthew effect and sample representativeness issues, future research should include municipal- and county-level local foundations. A comparative analysis between national and local foundations can reveal differences in high-performance configurational paths, as well as the moderating effects of regional philanthropic culture, government support and local institutional environment.

Fourth, classify foundations by type and explore political connections. Future studies can divide foundations into government-associated foundations and privately initiated foundations. This classification helps to reveal the differentiated role of political connections: whether political connections shift from peripheral conditions to core conditions in government-associated foundations, and whether the intensity of professional management and program activity intensity is further enhanced in privately initiated foundations. Meanwhile, research can explore the interactive effects between political connections and market resources.

Fifth, investigate failure avoidance in low-performance paths. Based on the observed causal asymmetry, future research should focus on failure modes and avoidance mechanisms. Specifically, low-performance paths characterized by complete digital transparency infrastructure + strong political connections + low program activity intensity and complete digital transparency infrastructure + strong political connections + low professional management can be analyzed. Through comparative case studies, researchers can identify strategies for performance improvement and develop early warning models to help foundations avoid configurational traps.

**Revisions in the Manuscript:** These revisions have been made in Section 5.3.

**Comment 5:**- Terminological Inconsistency
The manuscript lacks a standardized lexicon for its core variables and organization types.
-- Acronym Conflicts: The text uses "BPL" and "BBL" interchangeably to refer to the "Brand Building Level".
-- Naming Conventions: The authors alternate between "non-profitable organizations" and "nonprofit organizations," which should be standardized to the latter for international academic standards.
-- Variable Labels: "Government Resource Acquisition" is abbreviated as both "GRA" and "GRS" in different sections of the text.

**Response:** We sincerely appreciate the reviewer for carefully identifying the terminological inconsistency problems in the manuscript. We have conducted a full-text check and revised all non-standard terms, acronyms and variable labels to ensure uniformity and academic normalization.

First, based on the feedback from the first reviewer, we have replaced the level of brand building in the study with the intensity of project activities. We have now rewritten all “BBL” with “PAI” to eliminate abbreviation conflicts.

Second, in line with international academic norms, we have replaced all instances of "non-profitable organizations" with the standard expression nonprofit organizations.

Third, we have confirmed and unified the abbreviation of Government Resource Acquisition as GRA, and removed all incorrect uses of "GRS" in the text.

All the above revisions have been completed in the revised manuscript, which effectively enhances the standardization and rigor of the paper.

**Comment 6:** - Structural Redundancy
-- The paper repeats large blocks of information, which hinders the flow of the argument.
--- Repetitive Findings: The core results (the two paths to effectiveness) are described in nearly identical language in the Abstract, the Introduction, the Path Analysis, and the Conclusions.
--- Framework Justification: The explanation of the TOE framework is repeated across the literature review and the research design sections, rather than being introduced once and then applied.
**Response:** We sincerely appreciate the reviewer’s insightful suggestion regarding structural redundancy. We have thoroughly revised the manuscript to eliminate repetitive content and enhance the logical flow between sections.

1. Elimination of Redundant TOE Framework Explanation

We have revised and streamlined the Research Design section by removing repetitive expressions, reorganizing the sentence structure, and presenting the variables based on the TOE framework more concisely and logically. The revised content no longer duplicates the framework explanation and significantly improves readability

These revisions are presented in Section 3.2.3 of the revised manuscript. The specific modifications are as follows: To avoid an unfavorable situation where the number of condition combinations far exceeds the sample size, thereby failing to reflect the actual situation, the selection of samples and variables strictly adheres to the selection principles of the QCA analysis method. When the number of case samples ranges from 10 to 60, it is considered a medium sample size, and the optimal number of condition variables should be maintained between 4 and 10. Based on previous studies and the TOE (Technology‑Organization‑Environment) framework established in the literature review, and considering data availability and completeness, this study defines the outcome variable and condition variables as follows. The outcome variable is Fundraising Effectiveness (FE). The condition variables are categorized into three dimensions: Environmental dimension: Political Connection (PC), Government Resource Acquisition (GRA); Organizational dimension: Professional Management Level (PML), Program Activity Intensity (PAI); Technological dimension: Digital Transparency Infrastructure (DTI).

2. Streamlining of Core Findings

We have thoroughly revised the presentations of the main results in the Abstract, Introduction, and Results & Discussion sections to avoid identical wording and ensure distinct emphases:

The Abstract provides a concise, objective summary of core conditions and pathways. The explanation of the core findings in the abstract section has been changed to: Research has found that: (1) digital transparency infrastructure and program activity intensity are key conditions for improving the fundraising effectiveness of charitable foundations; the level of professional management and political connections exert important impacts on enhancing the fund-raising effectiveness of foundations, serving as important influencing conditions for the improvement of fundraising effectiveness. (2) There are two core paths for charitable foundations to achieve high fundraising benefits: one is a combination of strong program activity, robust digital transparency, and high political connections; the other is a combination of strong program activity, robust digital transparency, and high professional management. This study enriches and expands the theoretical connotation and research paradigm of charitable fundraising research and provides empirical evidence and policy guidance for promoting the sustainable and healthy development of charitable foundations.

The Results & Discussion emphasizes the interpretation, mechanism, and theoretical implications of the findings rather than simple repetition. The conclusion section's explanation of the core findings has been changed to: “Seeking expansion but lacking funds” has become an urgent bottleneck for charitable organizations to overcome. However, acquiring development resources and enhancing fundraising effectiveness requires these organizations to actively explore solutions across political, organizational, and social dimensions. Research findings indicate two successful pathways for charitable foundations to achieve high fundraising effectiveness: first, the combined effect of comprehensive digital transparency infrastructure, high project activity intensity, and strong political connections; second, the combined effect of comprehensive digital transparency infrastructure, high project activity intensity, and high professional management levels. Compared to other factors, comprehensive digital transparency infrastructure and high project activity intensity are the most critical factors for charitable organizations to successfully achieve high fundraising returns.

These revisions eliminate redundancy while preserving key information, improving the readability and logical flow of the manuscript.

**Revisions in the Manuscript:** These revisions have been made in Abstract and Section 5.1.

**Comment 7:** - Stylistic and Idiomatic Clarity
-- Several phrases appear to be literal translations of Chinese academic idioms, which can be confusing for an English-speaking audience.
--- Translation Artifacts, like "in a 'different paths leading to the same goal' manner" are refequently appearing - consider the replacement with a better technical word e.g. "equifinality".
--- Awkward Phrasings, like "non collection" of of high-efficiency fundraising - maybe "absence of high fundraising effectiveness" or "low-performance configurations" would do the better job, here?

**Response:**

We sincerely appreciate the reviewer’s professional suggestions on improving linguistic accuracy and idiomatic academic expression. We have carefully revised all non-native and awkward phrases throughout the manuscript to conform to standard academic English. 1. We have replaced all literal-translation phrases such as “different paths leading to the same goal” with the rigorous and widely accepted fsQCA term equifinality. 2. We have removed all awkward expressions including “non collection of high-efficiency fundraising” and replaced them with standard academic terms such as low fundraising effectiveness, low-performance configurations, and absence of high fundraising efficiency. These revisions have greatly improved the clarity, fluency, and academic standardization of the manuscript.

The specific modifications are as follows:

-At the end of the second paragraph of the Introduction: Against this backdrop, this study analyzes 54 national charitable foundations in China, employing the fuzzy-set Qualitative Comparative Analysis (fsQCA) method to break through the limitations of traditional linear analysis and explore the factors and configuration effects that influence fundraising effectiveness. The study addresses two core questions: (1) Which conditions are more critical for improving the fundraising effectiveness of charitable foundations? (2) What equifinal configurations promote high fundraising effectiveness in charitable foundations?

-The second paragraph of the introduction：This unbalanced development is inherently linked to the “causal asymmetry between high and low fundraising efficiency” identified in this study: the core advantages of top-tier foundations (intensity of program activities, digital transparency infrastructure) further strengthen their resource acquisition capacity, whereas the shortcomings of grassroots foundations (low intensity of program activities, inadequate professional management) are amplified by the Matthew effect, ultimately forming the causal asymmetry characterized by convergent high-efficiency configurations and divergent low-efficiency configurations.

-The third paragraph of the introduction：At the practical level, the study provides a clear roadmap for the development of charitable foundations. It helps them identify core elements and effective strategy combinations for improving fundraising effectiveness, improve their funding acquisition pathways, increase funding acquisition efficiency, and help foundations avoid falling into low-performance fundraising traps.

-Starting from the penultimate paragraph of 4.3 Configuration Path Analysis: Finally, further analysis was conducted on the absence of high fundraising effectiveness and the causal asymmetry of its influencing mechanisms in charitable foundations, exploring the “causal asymmetry” of the influencing mechanism.

-At the beginning of the first paragraph of the main conclusion: Resource scarcity amid expansion aspirations has become a critical bottleneck for charitable organizations to overcome.

-The third suggestion in the 5.1 Main Conclusion: Third, establish a transparent and traceable supervision system.

**Revisions in the Manuscript:** These revisions have been made in Section 1, Section 4.3 and Section 5.1.

**Comment 8:** A NOTE ABOUT APPLIED LANGUAGE IN GENERAL
Areas for Potential Refinement
While the language is strong, there are occasional instances of redundant or slightly awkward phrasing typical of translated academic prose, such as "in a 'different paths leading to the same goal' manner" or describing the "non collection" of high-efficiency fundraising. Some sentences are dense and could be further simplified to improve clarity for a broader non-academic audience.

**Response:** We sincerely appreciate the reviewer’s valuable and constructive suggestions on refining the applied language of the manuscript. We fully agree that streamlining redundant phrasing, revising awkward expressions from literal translation, and simplifying dense sentences will significantly enhance the readability of the paper while preserving its academic rigor, which is also in line with the publishing norms of PLOS ONE for a broad readership.

In response to these comments, we have conducted a comprehensive revision of the entire manuscript with a focus on language polish and readability improvement. The key revisions are as follows:

1. Replaced literal translated and awkward expressions: We substituted the non-standard literal phrase "in a 'different paths leading to the same goal' manner" with the rigorous fsQCA professional term equifinal configurations, and revised the awkward expression "non collection of high-efficiency fundraising" to the standard academic phrase absence of high fundraising effectiveness, which conforms to the expression habits of international academic papers.

2. Simplified dense and lengthy sentences: We split overly complex long sentences, reorganized sentence structures, and used punctuation such as em dashes to optimize logical expression, making the argumentation more concise and smooth (e.g., simplifying the lengthy description of the Matthew effect and causal asymmetry in the Introduction, and streamlining the dense sentences about digital transparency infrastructure’s role in the Conclusions).

3. Eliminated redundant wording and unified expression: We deleted repetitive modifiers and redundant verbs/phrases throughout the text, unified inconsistent word usages, and simplified cumbersome nominal structures to avoid the awkwardness of translated academic prose.

4. Balanced academic rigor and readability: On the premise of retaining professional terms and research logic, we adjusted the expression of some professional content to be more accessible, ensuring that the paper is not only academically rigorous but also easy to understand for a broader non-academic audience.

All the above language revisions have been fully implemented in the revised manuscript, and the overall fluency, clarity and standardization of the paper have been significantly improved. We are very grateful for the reviewer’s professional guidance on the language expression of the manuscript.

We would like to express our sincere gratitude to the editor and all reviewers for their professional, constructive and insightful comments on our manuscript. Your valuable suggestions have not only significantly improved the academic rigor, clarity and completeness of this paper, but also provided us with important guidance for future research. We have carefully addressed all the comments and revised the manuscript accordingly. We hope the revised version meets your expectations and look forward to your favorable consideration. Thank you again for your time and efforts in reviewing our work.

Sincerely,

Heran Zou

Wuhan Institute of Technology
